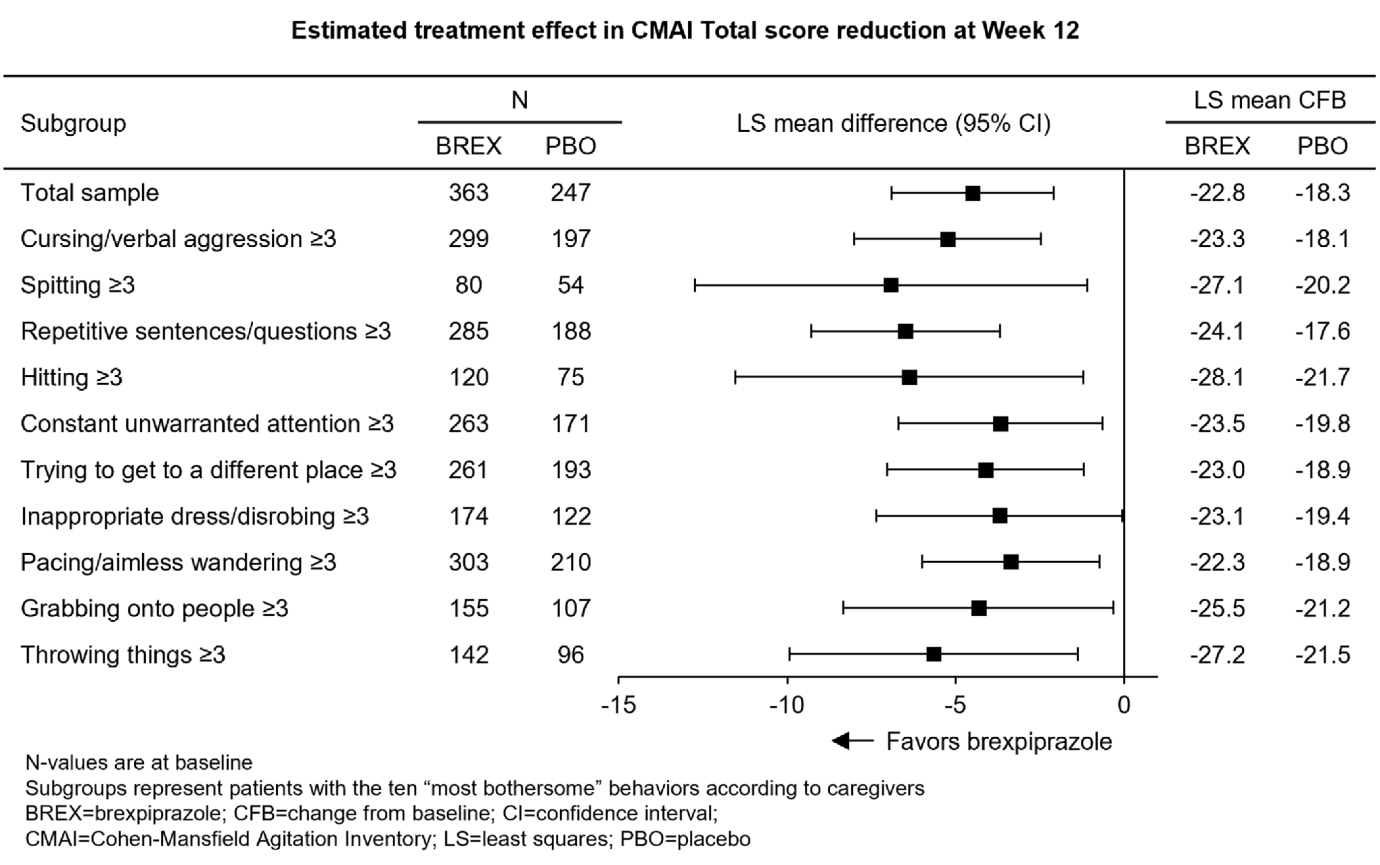# Efficacy of brexpiprazole on agitation in patients with dementia due to Alzheimer’s disease exhibiting behaviors most bothersome to caregivers: *post hoc* pooled analysis of two randomized controlled trials

**DOI:** 10.1002/alz.092333

**Published:** 2025-01-03

**Authors:** Malaak Brubaker, David Wang, Sanjeda R Chumki, Pedro Such, Zhen Zhang, Anton M Palma

**Affiliations:** ^1^ Otsuka Pharmaceutical Development & Commercialization Inc., Princeton, NJ USA; ^2^ Lundbeck LLC, Deerfield, IL USA; ^3^ H. Lundbeck A/S, Valby, Copenhagen Denmark

## Abstract

**Background:**

Agitation associated with dementia due to Alzheimer’s disease encompasses a wide range of behaviors, including excessive motor activity, verbal aggression, and physical aggression. In a survey of unpaid caregivers living with an individual with Alzheimer’s disease, the “most bothersome” agitation behaviors (which may influence the decision to transfer the patient to long‐term care) were: cursing or verbal aggression, spitting, repetitive sentences or questions, hitting, constant unwarranted requests for attention or help, trying to get to a different place, inappropriate dress or disrobing, pacing/aimless wandering, grabbing onto people, and throwing things. This *post hoc* analysis aimed to determine the efficacy of brexpiprazole on agitation in patients frequently exhibiting these bothersome behaviors.

**Method:**

Data were included from two 12‐week, randomized, double‐blind, placebo‐controlled, parallel‐arm trials of fixed‐dose brexpiprazole in patients with agitation associated with dementia due to Alzheimer’s disease (ClinicalTrials.gov identifiers: NCT01862640, NCT03548584). Efficacy was assessed using the Cohen‐Mansfield Agitation Inventory (CMAI), which comprises 29 agitation items scored from 1 (never occurs) to 7 (occurs a few times an hour). Least squares mean changes in CMAI Total score from baseline to Week 12 were determined in ten subsamples, each comprising patients for whom a specific “most bothersome” behavior occurred at least once per week (CMAI item score ≥3) at baseline. Data for brexpiprazole 2 or 3 mg/day (FDA‐approved dose) and for placebo were pooled.

**Result:**

The pooled sample comprised 610 patients (brexpiprazole, n = 363; placebo, n = 247). At baseline, the ten “most bothersome” agitation behaviors occurred at least weekly in subsamples ranging from 134 patients (spitting) to 513 patients (pacing/aimless wandering). At Week 12, in the ten subsamples, between‐group least squares mean differences in CMAI Total score change favored brexpiprazole (all p<0.05 versus placebo) (Figure). In 8/10 subsamples, greater improvement for brexpiprazole versus placebo was observed from (at least) Week 8 onwards.

**Conclusion:**

Agitation behaviors that are most bothersome to caregivers of patients with dementia due to Alzheimer’s disease (including excessive motor activity, verbal aggression, and physical aggression) were generally well represented in the brexpiprazole trials. In patients frequently exhibiting these bothersome behaviors, fixed‐dose brexpiprazole 2 or 3 mg/day was associated with larger improvements than placebo.